# Les plaies du tendon patellaire

**DOI:** 10.11604/pamj.2014.17.235.2527

**Published:** 2014-03-27

**Authors:** Atif Mechchat, Mohammed Elidrissi, Abdelhak Mardy, Abdelghni Elayoubi, Mohammed Shimi, Abdelhalim Elibrahimi, Abdelmajid Elmrini

**Affiliations:** 1 Service de Chirurgie Ostéoarticulaire B4, CHU Hassan II, Fès, Maroc

**Keywords:** Tendon patellaire, plaie, réparation primaire, patellar tendon, wound, primary repair

## Abstract

Les plaies du tendon patellaire sont peu fréquentes et sont peu rapportés dans la littérature, contrairement aux ruptures sous cutanées. Les sections du tendon patellaire nécessitent une réparation immédiate afin de rétablir l'appareil extenseur et de permettre une récupération fonctionnelle précoce. A travers ce travail rétrospectif sur 13 cas, nous analysons les aspects épidémiologiques, thérapeutiques et pronostiques de ce type de pathologie en comparant différents scores. L’âge moyen est de 25 ans avec une prédominance masculine. Les étiologies sont dominées par les accidents de la voie publique (68%) et les agressions par agent tranchant (26%) et contendant (6 %). Tous nos patients ont bénéficié d'un parage chirurgical avec suture tendineuse direct protégée par un laçage au fils d'aciers en légère flexion. La rééducation est débutée après sédation des phénomènes inflammatoires. Au dernier recul les résultats sont excellents et bon à 92%. Nous n'avons pas noté de différence de force musculaire et d'amplitude articulaire entre le genou sain et le genou lésé. Les lésions ouvertes du tendon patellaire est relativement rare. La prise en charge chirurgicale rapide donne des résultats assez satisfaisants. La réparation est généralement renforcée par un semi-tendineux, synthétique ou métallique en forme de cadre de renfort pour faciliter la réadaptation et réduire le risque de récidive après la fin de l'immobilisation.

## Introduction

Les sections du tendon patellaire sont rares et nécessite une réparation immédiate afin de rétablir la continuité de l'appareil extenseur et de permettre une récupération fonctionnelle active précoce [1–4]. Le but de ce travail est d'analyser l'aspect épidémiologique de cette pathologie et d’évaluer les résultats cliniques de la réparation chirurgicale en se basant sur plusieurs scores fonctionnels.

## Méthodes

Il s'agit d'une étude rétrospective de 13 cas de section totale du tendon patellaire colligés sur une période 4 ans de janvier 2009 à décembre 2012 avec un recul moyen de 18 mois. L’âge moyen de nos patients est de 25 ans avec des extrêmes allant de 18 à 31 ans. Le sexe masculin prédominait avec neuf hommes et deux femmes. Les étiologies étaient dominées par les accidents de la voie publique 68% et les agressions par agent contendant 26%.le coté droit étaient atteint dans 07 cas 72%. L'agent vulnérant était un objet tranchant dans 26% et contendant dans 6% des cas. La plaie était linéaire à bord déchiquetés dans 11 cas et largement contuse dans 2 cas. L'examen clinique avait retrouvé déficit d'extension active du genou. La prophylaxie antitétanique est systématique et une antibioprophylaxie à base de C2G avait été démarré dès l'admission et continuer pendant 48H. L'exploration chirurgicale était systématique La rachianesthésie était utilisé dans 100% des cas.

Le suivi est assuré par deux médecins juniors (E.M) et (B.H) incluant un examen clinique des deux genoux, mesure de la force musculaire et deux scores spécifiques : turba score [5], et insall score [6]. Tous les patients ont bénéficiés d'un traitement chirurgical. Nous avions 09 cas de section au niveau de la partie moyenne du tendon 03 cas de section proximal et un cas d'avulsion distal emportant la tubérosité tibiale antérieur.

### Technique chirurgicale

**Figure 1:**
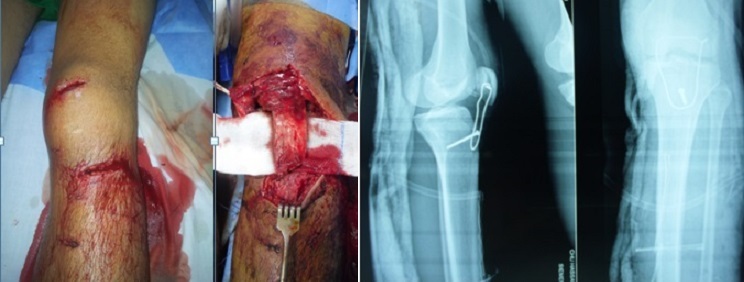
Section distale du tendon patellaire avec arrachement osseux de la tubérosité tibiale ayant nécessité un vissage associé a un cerclage

**Figure 2:**
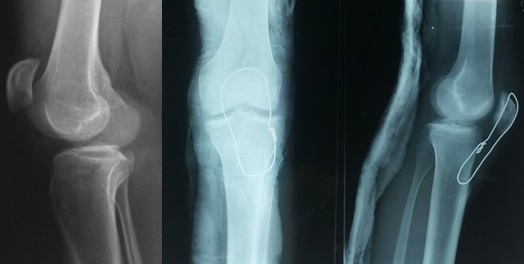
Cerclage entre la patella et le tibia dans le cadre d’une section du 1/3 moyen du tendon patellaire

Le premier temps opératoire a consisté en un parage chirurgical. L'abord avait été fait par un élargissement de la plaie par une incision antérieur médiane. Le tendon était sectionne dans 81 % des cas (Figure 1, Figure 2), et partiellement dans 2 cas. Dans un cas nous avions une effraction capsulaire avec section du pivot centrale sans lésion méniscale. Pour tous les tendons nous avons procédé à une régularisation économique des berges puis suture directe par un laçage à l'aide d'un fils a résorption lente 2 mm renforcé d'un surjet au fils résorbable plus fin 4/0 avec suture de la gaine du tendon. Nous avons protégé nos sutures par un laçage au fils d'acier 18/10 en légère flexion à 30 °que nous avons enlevé à la 6 ème semaine.

### Les suites post opératoires

Le quadriceps est un muscle très puissant, et les sutures peuvent se casser sous une contraction soudaine, comme, par exemple, pour empêcher soi-même de tomber ou de trébucher. Une Immobilisation stricte du genou dans une botte de marche est recommandée pour au moins 1 mois, suivie d′une attelle amovible jusqu′à la réhabilitation de la flexion passive sur une attelle motorisée, suivie par un travail excentrique du quadriceps en position couchée et, enfin, par un travail concentriques. L′attelle motorisée est retiré une fois l'extension active est terminée, avec une bonne contraction du quadriceps (souvent, en 2 ou 3 semaines). Quatre à 6 mois sont généralement nécessaire pour récupérer la fonction du genou normal.

## Résultats

Les résultats ont été appréciés après un recul moyen de 18 mois. Le Tableau 1 présente les résultats de l'examen clinique et des différents scores. Nous n'avons pas trouvé de différence (p > 0.05) entre le secteur de mobilité et la trophicité musculaire par rapport au coté sain. L'examen clinique n'a pas montré de patella baja ou alta lors du suivi. Les suites post opératoire sont marquées par un seul cas de sepsis superficielle jugulé par des soins locaux et antibiothérapie et trois cas d'amyotrophie sans retentissement fonctionnel et un cas de syndrome neuroalgodystrophique.

**Tableau 1:** Résultats clinique et scores au dernier recul des patients

Score	Intervalle	Résultats (intervalle)
Age		25(18- 31)
Douleur	0-10	1.2 (0- 5)
Force non opéré		27.1 (12- 37)
Force opéré		25.4 (6- 36)
Circonférence non opéré		42.6 (33.5- 49)
Circonférence opéré		42.1 (33- 49)
Secteur mobilité opéré		131.3 (105- 150)
Secteur mobilité non opéré		134.7 (110-150)
Turba objectif	0-14	2.6 (0-5)
Turba subjectif	0-12	2.1 (0-5)
Insall genou	0-100	91.9 (80-100)
Insall fonction	0-100	90.3 (60-100)
Satisfaction	1-4	3.4 (2-4)

## Discussion

Presque toutes les séries de la littérature englobe rupture sous cutanée et plaie du tendon patellaire avec un faible pourcentage de ces derniers [7, 8]. Le caractère ouvert de la lésion pousse effectivement les patients a consulté par contre lorsque la rupture est sous cutanée la lésion peut passer inaperçu et peut rentrer dans la chronicité. Une rupture récente du tendon patellaire date de moins de 3semaines. Ces lésions surviennent le plus souvent avant 40 ans (98% des cas) dans la série de Siwek et Rao [9]). Nos patients sont jeunes avec un âge moyen de 25 ans.il s'agit donc d'une pathologie de la population active. Ces lésions surviennent préférentiellement chez l'homme [9].. Le diagnostic est volontiers clinique, suspecté par le siège de la lésion devant le tendon patellaire avec un déficit de l'extension active du genou, mais il peut passer inaperçu dans le cadre des urgences, en particulier si la rupture est partielle La lésion la plus fréquente est, selon Lindy et al. [10], la désinsertion rotulienne suivie de la rupture en plein corps. Dans notre contexte la lésion au 1/3 moyen du tendon est la plus fréquente suivie de la désinsertion proximale. Nous ne pratiquons pas de bilans paraclinique en dehors d'une radiographie standard à la recherche de lésions associé, de corps étranger radio-opaque, ou de signes indirects d'effraction capsulaire tel que la présence d'air en intra-articulaire.

La prise en charge est celle de tout traumatisme ouvert de membre, la prophylaxie antitétanique et l'antibiothérapie sont systématiques. Nous utilisons une céphalosporine deuxième génération débutée à l'admission est poursuivie pendant 48h. Pour tous les patients vu au delà de 6h nous prolongeons l'antibiothérapie à 10 jours en association a un aminoside +/- imidazolé.

L'intervention commence dans tous les cas par un parage chirurgical suivi du rétablissement de la continuité du tendon. Dans le cadre de patients vus tardivement avec une plaie souillé, saillant et coll [11], recommande la réparation en deux temps afin d'éviter les désunions par sepsis. Tous nos patients ont bénéficé de technique de suture termino-terminale renforcé par un surjet péritendineux et protégé par un laçage par fils d'acier entre la patella et le tibia.la fermeture de la gaine du tendon est capitale car elle conditionne la qualité des résultats au long cours [12]. Les modalités techniques sont variables selon le type de rupture et selon les auteurs. Il y a les sutures simples protégées par un cerclage métallique proposé par Mc Laughlin en 1956 et repris par de nombreux auteurs [13, 14]. Pour obtenir d'emblée une réparation solide, compatible avec une rééducation précoce, sans avoir recours au cadrage métallique, certains auteurs utilisent une autogreffe pour réaliser le cerclage qui sert également de plastie tendineuse. Certains utilisent le DIDT [15], d'autres [1] une autogreffe de demi-tendineux et un renfort central constitué par le retournement sur lui-même du surtout prérotulien. Pour la plupart des auteurs, la suture des ailerons doit être systématiquement réalisée [16]. Selon Ait Si Selmi et al. [17], une radiographie de contrôle de profil à 30° doit toujours être réalisée après réparation du tendon pour régler la hauteur patellaire par comparaison au côté sain (index de Caton et Deschamps = 1±0,2).

La rééducation a une place primordiale dans la récupération fonctionnelle du membre [18]. L'appui complet était autorisé à j1 et protégé par une attelle en extension entre les séances de kinésithérapie. La rééducation dans un premier temps devrait être axée sur la récupération des amplitudes articulaires initialement passives et limitées à 90° de flexion pendant les 45 premiers jours, l'extension active était proscrite. À partir de la sixième semaine les amplitudes de la mobilisation étaient progressivement augmentées et le quadriceps était renforcé. Le faible nombre de complication en postopératoire est due aux mesures d'hygiène strict avec une attelle pendant 6 semaines.

## Conclusion

Les lésions ouvertes du tendon patellaire est relativement rare. La prise en charge chirurgicale rapide donne des résultats assez satisfaisants. La réparation est généralement renforcée par un semi-tendineux, synthétique ou métallique en forme de cadre de renfort pour faciliter la réadaptation et réduire le risque de récidive après la fin de l'immobilisation.
